# A multievent approach to estimating pair fidelity and heterogeneity in state transitions

**DOI:** 10.1002/ece3.729

**Published:** 2013-10-04

**Authors:** Antica Culina, Shelly Lachish, Roger Pradel, Remi Choquet, Ben C Sheldon

**Affiliations:** 1 Department of Zoology, Edward Grey Institute, University of OxfordSouth Parks Road, Oxford, OX1 3PS, U.K; 2 Centre d'Ecologie Fonctionnelle et Evolutive, Campus CNRS, UMR51751919 Route de Mende, F-34293, Montpellier, Cedex 05, France

**Keywords:** Great tit, heterogeneous recapture rates, multievent mark–recapture modeling, survival

## Abstract

Fidelity rates of pair-bonded individuals are of considerable interest to behavioral and population biologists as they can influence population structure, mating rates, population productivity, and gene flow. Estimates of fidelity rates calculated from direct observations of pairs in consecutive breeding seasons may be biased because (i) individuals that are not seen are assumed to be dead, (ii) variation in the detectability of individuals is ignored, and (iii) pair status must be known with certainty. This can lead to a high proportion of observations being ignored. This approach also restricts the way variation in fidelity rates for different types of individuals, or the covariation between fidelity and other vital rates (e.g., survival) can be analyzed. In this study, we develop a probabilistic multievent capture–mark–recapture (MECMR) modeling framework for estimating pair fidelity rates that accounts for imperfect detection rates and capture heterogeneity, explicitly incorporates uncertainty in the assessment of pair status, and allows estimates of state-dependent survival and fidelity rates to be obtained simultaneously. We demonstrate the utility of our approach for investigating patterns of fidelity in pair-bonded individuals, by applying it to 30 years of breeding data from a wild population of great tits *Parus major* Linnaeus. Results of model selection supported state-dependent recapture, survival, and fidelity rates. Recapture rates were higher for individuals breeding with their previous partner than for those breeding with a different partner. Faithful birds that were breeding with the same partner as in the previous breeding season (i.e., at *t* − 1) experienced substantially higher survival rates (between *t* and *t* + 1) and were also more likely to remain faithful to their current partner (i.e., to remain in the faithful state at *t* + 1). First year breeders were more likely to change partner than older birds. These findings imply that traditional estimates, which do not account for state-dependent parameters, may be both inaccurate and biased, and hence, inferences based on them may conceal important biological effects. This was demonstrated in the analysis of simulated capture histories, which showed that our MECMR model was able to estimate state-dependant survival and pair fidelity rates in the face of varying state-dependant recapture rates robustly, and more accurately, than the traditional method. In addition, this new modeling approach provides a statistically rigorous framework for testing hypothesis about the causes and consequences of fidelity to a partner for natural populations. The novel modeling approach described here can readily be applied, either in its current form or via extension, to other populations and other types of dyadic interactions (e.g., between nonpaired individuals, such as parent–offspring relationships, or between individuals and locations, such as nest-site fidelity).

## Introduction

The pair bonds formed between males and females for the purpose of reproduction are among the most widely studied types of associations between two individuals. In birds, a group where more than 85% of species are socially monogamous (Bennett and Owens 2002), the degree to which pair bonds are maintained over consecutive breeding seasons (i.e., the pair fidelity rate) is known to be highly variable both within and between species (Black 1996). Explaining why these differences occur is important for understanding the evolution of social monogamy and long-term partnerships (reviewed in Black 1996; Reichard and Boesch 2003; Shuster and Wade 2003). Moreover, the dynamics of the formation and maintenance of pair bonds can influence population productivity and hence shape population dynamics, both through determining the number of reproductive pairs (Sugg et al. 1996; Berec and Boukal 2004; Maxin and Berec 2010), and through differences in reproductive success of newly formed and existing pairs (e.g., Pampus et al. 2005; Hatch and Westneat 2008).

Despite the importance of understanding the dynamics of pair bonds, obtaining accurate estimates of pair fidelity in wild populations remains a significant challenge. Existing estimates have been obtained from observations of marked individuals that are recaptured (or resighted) in consecutive breeding seasons (e.g., Rogers and Knight 2006; Hatch and Westneat 2008). The principal drawback of this approach is that low recapture rates (a common problem for many taxa, see in Archaux et al. 2012) can bias estimates of fidelity because a proportion of the marked individuals that are alive will not be recaptured at a particular sampling occasion. Moreover, recapture probabilities may differ among different classes of individuals because of underlying biological, ecological, or behavioral characteristics (e.g., males and females, paired and unpaired individuals; Crespin et al. 2008; Carter et al. 2012 and references therein). When such differences in detectability are ignored, biologically important effects that operate on the trait of interest may be concealed due to biases in rate estimates, and inferences based on observed patterns will be artifacts of individual encounter rates (Cubaynes et al. 2010; Carter et al. 2012; Fletcher et al. 2012).

An additional, and interrelated, problem in estimating pair fidelity rates from field observations is the inherent difficulty associated with assigning pair status with certainty. Determining whether a focal individual has been faithful to its partner from the previous season requires that both its current and previous partners are known and captured. For this to occur a large proportion of the population must be marked, and recapture rates for marked individuals must be high. As discussed above, this requirement is rarely met in studies of wild populations, and consequently, most estimates of pair fidelity rates are obtained after a substantial part of the data has been discarded (i.e., Forslund and Larsson 1991; Warkentin et al. 1991; Llambias et al. 2008).

The estimation of association rates in other types of dyadic interactions (such as associations between individuals in social network, Croft et al. 2008), where the nature or strength of association is determined from field-based observations of marked individuals could also suffer from these same problems (e.g., Voelkl et al. 2011). Therefore, if we want to robustly quantify rates of maintenance of pair bonds and other types of dyadic interactions, a modeling framework that incorporates and accounts for both imperfect and heterogeneous recapture rates, and problems connected with state assignment is needed. Such a framework would not only provide a robust method for estimating rates but would, at the same time, allow greater flexibility to test hypotheses on causes, and fitness costs and benefits of maintenance of pair bonds (e.g., the survival consequences of fidelity and mate change; see below).

Multistate capture–mark–recapture (MSCMR) is a modeling framework widely used to estimate state-dependent demographic rates (commonly survival rates) along with transition rates of individuals among different “states” (e.g., physical locations, infection status, reproductive status; Nichols et al. 1994; Lebreton and Pradel 2002), while explicitly accounting for imperfect and heterogeneous detectability of marked individuals (Arnason 1972, 1973; Hestbeck et al. 1991). As it is possible to assign individuals to different states based on their pair status (with the same partner or not), multistate models provide a framework for estimating pair fidelity rates and survival probabilities simultaneously in the same model. Moreover, by allowing different constraints to be imposed on state-dependent survival and transition parameters, these models provide a rigorous method of evaluating the fitness consequences of pair fidelity. One drawback of MSCMR models that limits their utility is that they assume there is no error or ambiguity in state assignment, an assumption rarely met in studies of pair fidelity (or other types of dyadic interactions). Recently developed multievent capture–mark–recapture models (MECMR) (Pradel 2005), however, provide an extension of the MSCMR models that explicitly accounts for unknown or partially observable states by treating them as a hidden Markov process (Pradel 2005; Rouan 2007). In MECMR models, observations of captured individuals, and indirect information from individuals that are not captured (globally termed “events”), are related to the true but unknown (hidden) state of the individual through a series of conditional probabilities (Pradel et al. 2008; Choquet et al. 2009b). Accordingly, MECMR models offer an ideal framework to obtain robust estimates of pair fidelity when there is ambiguity of state assessment.

In this paper, we present a flexible MECMR modeling framework to obtain robust estimates of pair fidelity in wild populations. We based the conceptual development of our modeling approach on the pair bond between the members of monogamous breeding pairs (where females and males exclusively associate together at the breeding site) because this is one of the most studied dyadic interactions in behavioral, population, and evolutionary ecology. Also, the way that data on pair memberships is collected is similar to the way in which information on other dyadic associations in wild are obtained.

To demonstrate the utility of our approach, we apply it to 30 years of data from a wild population of socially monogamous great tits *Parus major* Linnaeus, to estimate annual survival and fidelity rates and investigate patterns of pair fidelity in the population. We demonstrate the utility and advantages of our new modeling approach by combining the results of MECMR modeling with model selection to test the following three hypotheses: (a) pair fidelity confers benefits to individuals in terms of enhanced survival (e.g., because greater familiarity and better coordination between partners affords paired individuals an enhanced physical condition [Hall 1999; Black et al. 2007; Naves et al. 2007], while when survival is related to social rank in winter flocks, such as occurs in the great tit, one sex will benefit by pairing with a dominant partner [Ekman 1990; Lemmon et al. 1997]); (b) adults have higher pair fidelity rates (i.e., the probability of remaining faithful to last year's partner) than do yearlings (e.g., Ens et al. 1993; Choudhury 1995; Pampus et al. 2005; Llambias et al. 2008); and (c) individuals that were faithful to their previous partner have higher pair fidelity rates than those that changed partner because birds that have found the best possible partner, either in terms of quality (“better option” hypotheses, Ens et al. 1993), or compatibility (“compatibility hypothesis,” Choudhury 1995) should tend to stay with them. We then use simulated capture histories with known parameter values to test the model performance under different values of state-dependent recapture rates. Finally, we demonstrate that this modeling framework is readily applicable to other dyadic interactions, and consider several possible extensions and applications of the framework, as well as discussing its limitations.

## Methods

### Multievent model framework for estimating pair fidelity

**Figure 1:**
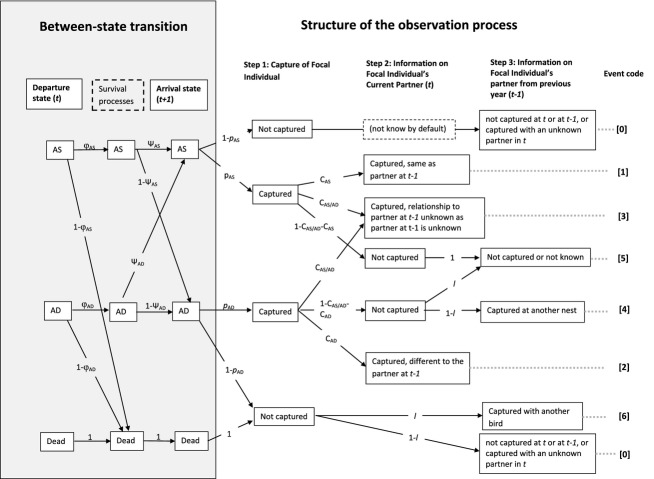
Illustrative figure of the between-state transition process and the structure of the observation process used to estimate pair fidelity rates. Three possible states are “AS,” alive and breeding with the previous year partner; “AD,” alive and breeding with a partner different to the previous year one; “D,” dead (cannot appear for the initial capture of individuals). Between-state transition process has two steps: survival (φ_AS_, survival probability for individuals in the state “AS”; φ_AD_, survival probability for individuals in the state “AD”) and transition among live states, conditional on survival (ψ_AS->AS_, probability of staying with the same partner for individuals in the state “AS”; ψ_AD->AS_, probability of staying with the same partner for individuals in the state “AD”). Observation process is composed of three steps: capture of the focal individual, information on focal individual's current partner, and information on focal individual's previous year partner. Event codes used to construct the capture histories of focal individuals are given in squared brackets. Probability parameters are given above the arrows and explained in more detail in the “Methods” section.

In MECMR models, individuals move independently among a finite set of mutually exclusive states, over a finite set of capture occasions (Pradel 2005). At each capture occasion, an event is observed. Events are related to the true, but not necessarily known, state of the individual through a series of conditional probabilities (Pradel et al. 2008; Choquet et al. 2009b). The general MECMR model for estimating pair fidelity we developed here uses three exclusive states: (1) Alive with the same partner (state “AS”): the focal individual is alive and breeding with its partner from the previous year; (2) Alive with a different partner (state “AD”): the focal individual is alive and breeding with a different partner to the previous year; and (3) Dead (state “D”): the focal individual is dead (this state is unobservable, but explicitly included in all multievent models). An individual can occupy only one state in a given breeding season. Transitions among these three states (i.e., “AS”, “AD”, “D”) are modeled as a two-step process composed of the probability of survival over the annual time interval, followed by the probability of transitioning among live states (i.e., transitions among states are conditional on survival over the time period, see Fig. 1).

The capture histories of individuals are coded as a series of observed events (Fig. 1). Events contain information on the capture of the focal individual combined with information on its pair status, which is in turn determined using additional information on the capture histories of current and previous partners of the focal individual, as well as knowledge of their pair status. Using this information, we defined seven exclusive events that could be observed at each capture occasion:

- event 0 = the focal individual is not captured in the current breeding season (i.e., at *t*), its partner from the previous season (*t* − 1) is either not captured at *t* − 1 or *t*, or is captured breeding at *t* at an active nest with an unknown partner;
- event 1 = the focal individual is captured at *t*, and is breeding with its partner from *t* − 1;
- event 2 = the focal individual is captured at *t* but is breeding with a different partner to that from *t* − 1;
- event 3 = the focal individual is captured at *t* but it is not known whether its current partner, which is captured, is the same as the one from *t* − 1;
- event 4 = the focal individual is captured at *t*, its current partner is not captured, but its partner from *t* − 1 is captured at *t* at a different nest (and hence is not breeding with the focal individual at *t*);
- event 5 = the focal individual is captured at *t*, its current partner is not captured, and its partner from *t* − 1 is either not captured in *t* or was not known in *t* − 1;
- event 6 = the focal individual is not captured at *t* (and hence its current partner is unknown), but its partner from *t* − 1 is captured breeding with another individual at *t*.

These seven events are the most general case of event construction for this type of dyadic association. Events 1, 2, and 4 are possible only under a single underlying state, while the remaining events are possible under multiple states (see Fig. 1 for further details).

### Specification of parameters and the model structure

Following notation in Pradel (2005) our model is defined with three types of parameters: (1) initial state probabilities π, represented in a vector of probabilities, Π; (2) transition probabilities involving: survival probabilities, ϕ (represented in the survival matrix, Ф), and between-state transition probabilities, ψ (conditional on survival, and represented in the transition matrix, ψ); and (3) event probabilities. Event probabilities are conditional on the underlying state, that is, *p*(event | state), and are composed of (a) the recapture probability of the focal individual *p* (i.e., *p*(focal capture status | state), represented in the matrix, P); (b) the capture probability of its current partner *c*, conditional on the capture of the focal individual (i.e., *p*(focal partner status | focal capture status), represented in the matrix, P2); and (c) the capture probability of the previous year's partner *l*, incorporating knowledge of its pairing status in the current year (*p*(event | focal partner status), represented in the matrix, P3). These three event matrices connect an observed event with the underlying state, such that:


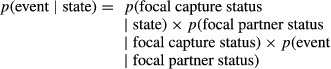
(1)

or, alternatively


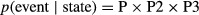
(2)

The vector of initial state probabilities (eq. 3) is composed of two elements representing two possible states (“AS” or “AD”) at the time of first capture of the individual. The state dead is not possible at the first capture, and thus has probability zero.


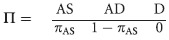
(3)

The rows of the survival matrix (eq. 4) specify the state (“AS” or “AD”) an individual is at the time *t*, as indicated in the first column. The elements of this matrix represent the probabilities that an individual in a given state at time *t* will survive (ϕ_AS_ is survival probability of an individual in the “AS” state and ϕ_AD_ of individual in the “AD” state) or die (1 − ϕ_AS_, 1 − ϕ_AD_) between *t* and *t* + 1, with the arrival state (“AS,” “AD,” “D”) indicated in the row above the matrix.


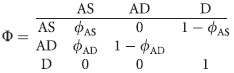
(4)

The rows of the transition matrix (eq. 5) specify the state an individual is at time *t* (as indicated in the first column), while the columns specify its state at time *t* + 1 (as indicated in the row above the matrix). The elements of this matrix are transition probabilities from states at time *t* to states at time *t* + 1, conditional on the survival over the time period.


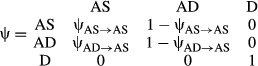
(5)

Rows of the matrix P (eq. 5) correspond to the columns of the between-state transition matrix, and represent the state an individual is in at *t*. Columns of this matrix define whether the focal individual is captured (FC) or not (FNc) given its underlying state (indicated with a superscript after FC and FNc). Thus, *p*_AS_ and *p*_AD_ are capture probabilities for individuals in the state “AS” or “AD,” respectively.


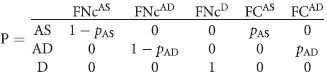
(6)

The matrix P2 (eq. 6) describes the combined probability of capturing the focal individual's current partner and knowing its identity in relation to the focal individual's previous partner (i.e., same as, or different to previous partner), conditional on the capture of the focal individual. The rows of matrix P2 correspond to the columns of matrix P. Elements of the matrix P2 define probabilities that the current partner is captured (PC) or not captured (PNc), or whether the current partner is captured but it is not known if it is the same or different to the one in the last year (PNn). Superscripts specify the underlying state of the focal individual. 1−∑*C* represents the complement to the sum of other parameters in the same row. Columns PNc^AS^ and PNc^AD^ appear in the matrix twice, because the two on the left side of the matrix correspond to capture of the current partner when the focal is not captured (and this equals 1), while the two on the right side correspond to the capture of the current partner when the focal is captured. It was necessary to structure P2 matrix in this way to allow the construction of the P3 matrix.


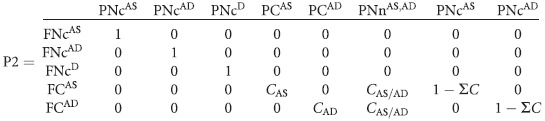
(7)

Finally, matrix P3 (eq. 8) describes the probability of capturing the focal individual's partner from the previous breeding season (*t* − 1) in the current capture occasion (*t*), incorporating information on its pairing status at time *t*. Note that if the focal individual's current partner was breeding with another individual than the focal at *t* − 1, then this indicates partner change. The rows of matrix P3 correspond to the columns of matrix P2. The column numbers correspond to the event codes (see above and Fig. 1).


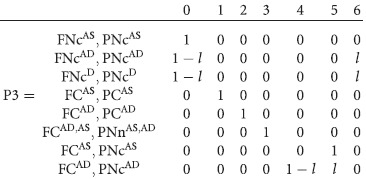
(8)

### Application of the MECMR to great tit breeding data

To demonstrate the utility of this MECMR modeling framework for estimating pair fidelity rates, we applied the model to 30 years of breeding records (1980–2009) from a long-term monitored population of great tits breeding in nest boxes. Around 1020 nest boxes are distributed at variable densities within the c. 385 ha mixed deciduous woodland of Wytham Woods, Oxfordshire, U.K. During the synchronous breeding season (April–May), nest boxes were visited on a weekly basis to collect data on breeding attempts. Individuals were captured between days 6 and 14 of the nestling phase either within the nest box by hand or using traps, or with mist-nets in front of the box. All captured individuals and all nestlings that survived to day 15 were marked with unique metal rings. The sex of parents was determined based on the presence (female) or absence (male) of a brood patch. Age (either yearling or adult, 2+ years old) was determined from plumage characteristics (Svensson 1992) or ringing records.

Capture histories for individuals (coded according to the seven events described above) were obtained for 4784 females and 4430 males, comprising 7837 breeding attempts. Both parents were captured in 84.5% of breeding attempts. In 12.9% of breeding attempts only the female was captured, and in the remaining 2.6% only the male was captured. To avoid issues of statistical nonindependence in analyses, the MECMR framework was applied to capture histories of males and females separately. MECMR models were built in E-SURGE Version 1.8.5 (Choquet et al. 2009b; Choquet and Nogue 2011). Implementation details are given in the Data S1 (Section 1). We assessed the identifiability of this model in E-SURGE with simulated datasets and with our data according to methods described in Choquet and Cole (2012). To ensure convergence of models on the global minima, models were run using repeated random initial values.

### Model covariates and model selection process

Currently, there is no specific goodness-of-fit (GOF) test for MECMR models. Thus, we assessed the fit of the general mark–recapture assumptions to our data by assessing the GOF of the single state Cormack–Jolly–Seber (CJS) model (Cormack 1964; Jolly 1965; Seber 1965), which allows for full time variation and no age effect in survival and recapture rates (Choquet et al. 2009a). The CJS model assumes all animals present at the same sampling occasion have equal future survival and recapture probabilities regardless of past history and capture in the current sampling occasion. These assumptions were tested using program U-CARE (Choquet et al. 2005, 2009a).

Starting models in both analyses (for males and females) allowed for time and state dependence in recapture probability of the focal individual, and for state, time, and age (yearlings and adults) dependence in survival and transition probabilities. Variation by state was restricted to adults only, as yearling birds have never previously bred and their survival and transition rates cannot depend on their current pair status. The initial state for all individuals was arbitrarily chosen to be “AD,” and fixed accordingly.

We used a 3-stage model selection process (Grosbois and Tavecchia 2003). First, we modeled recapture rates as constant, or as varying in relation to state, or time (yearly variation), while keeping survival and transition rates fully parameterized. Next, we used the best recapture rate model identified in the first stage (i.e., the model with the lowest Akaike Information Criteria [AIC]), to model survival rates as constant, or as varying in relation to state, age, and time. Finally, with recapture and survival rates parameterized according to the best models identified above, we modeled transition rates as constant, or varying in relation to age, time, and departure state (the state an individual transitions from). We considered additive effects of all these variables (time, age, state) on rate parameters and included interactions where we considered that these were biologically sensible (see Table S1). In total we performed model selection on a candidate list of four different recapture, ten different survival, and eleven different transition rate models (see Table S1 for full listing). Model selection was based on AIC (Anderson and Burnham 2002). Normalized AIC weights (w_*i*_) were used as a measure of relative support for each model.

### Evaluating model performance on the simulated capture histories

We evaluated the ability of our new modeling framework to estimate state-dependent survival and fidelity rates, under varying recapture rates using simulated capture histories. We also compared the accuracy of the pair fidelity rates obtained by our new model to those obtained by the traditional method (which ignores imperfect detectability and capture heterogeneity and where only definitive cases of fidelity and partner change can be considered). We simulated three separate capture history datasets consisting of 6400 capture histories, over 10 sampling occasions, under state-dependent but varying recapture rates (recapture probabilities of birds in the state “AD” for three simulations were 0.5, 0.7, and 0.8, while the recapture rate for birds in state “AS” was 0.9). The values of all other parameters were chosen to reflect the values obtained from our great tit dataset (ignoring temporal variation), and were kept the same in all three simulations (φ_AS_ = 0.6, φ_AD_ = 0.4; ψ_AS_ = 0.4; ψ_AD_ = 0.3; *c*_AS_ and *c*_AD_ = 0.73; *c*_AS/AD_ = 0.13, *l* = 0.92).

To estimate parameters using the MECMR modeling framework, we ran a model in which survival and transition rates were dependent on the state of departure, with recapture rates dependent on the state of arrival. To calculate pair fidelity rates using the traditional method, we applied the following equation:


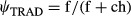
(9)

where f is the number of times event 1 occurred (definite case of pair fidelity) and ch is the number of times events 2 and 4 occurred (definite cases of partner change). To calculate pair fidelity rates of birds breeding with the same partner as in *t* − 1 we applied equation 9 to birds that were recorded in event 1 at *t* and in events 1, 2, or 4 at *t* + 1. To calculate pair fidelity rates of birds breeding with a different partner to that at *t* − 1, we applied equation 8 to birds that were recorded in events 2 or 4 at *t* and in events 1, 2, or 4 at *t* + 1.

## Results

The results of the GOF tests revealed no detectable lack of fit of the CJS model in either dataset (females: χ^2^ = 80.10, df = 91, *P* = 0.786; males: χ^2^ = 76.14, df = 99, *P* = 0.957), indicating that the general assumptions of the CJS model were reasonably met for both datasets. In addition there was no evidence for overdispersion in either dataset (females: ĉ = 0.88; males: ĉ = 0.88).

**Figure 2:**
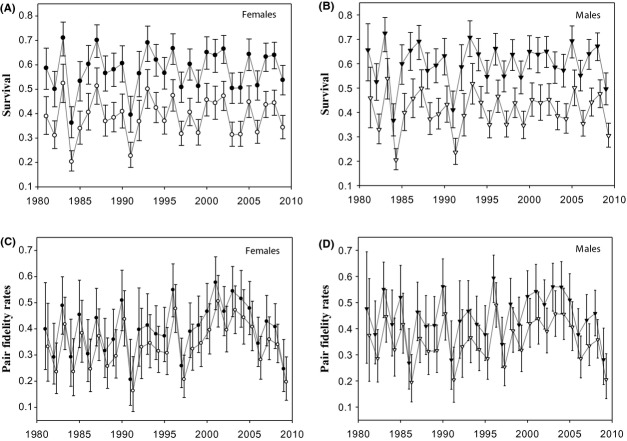
Survival rates (±95% CI) for (A) adult female and (B) adult male great tits by pair status (estimates obtained from best supported model for each sex, see Tables 1 and 2) and pair fidelity rates (±95% CI) for adult (C) female and (D) male great tits (estimates obtained from best supported model for both sexes, see Tables 1 and 2). Filled symbols show rates for individuals that remained with their previous partner (state “AS”) while open symbols show rates for individuals that changed partners (state “AD”).

For both sexes, there was strong support for models in which recapture rates were constant over the study period but varied by state (i.e., pair status, Tables 1 and 2). Individuals that were faithful to the previous year's partner were more likely to be recaptured than were individuals that had changed partners (recapture rate: faithful females = 0.72, CI = 0.69–0.76, changed partner females = 0.65, CI = 0.63–0.67, faithful males = 0.61, CI = 0.57–0.64, changed partner males = 0.55, CI = 0.53–0.57, Fig. S1). For both sexes, the best supported model in the candidate set allowed survival rates to vary with time, age, and by state (i.e., according to pair status of adults, Tables 1 and 2). As predicted, birds that remained with their previous partner had higher annual survival rates than birds that had changed partners (Fig. 2A and B). For both sexes, the best supported transition rate model allowed annual pair fidelity rates to differ between adults and yearlings, and to vary over time (Tables 1 and 2). Importantly, this best supported model also allowed transition rates to differ according to the pair status of adult birds (Tables 1 and 2). Adults that had bred with their current partner in the previous year displayed greater pair fidelity rates than did adults that had not bred with their current partner in the previous year (i.e., the probability of transitioning from state “AS” to state “AS” was higher than the probability of transitioning from state “AD” to “AS,” Fig. 2C and D).

**Table 1:** Summary results of the multievent mark–recapture analysis to estimate recapture, survival, and pair fidelity rates in female great tits

	Model Structure							
						
Parameter	p	φ	ψ		dev	AIC	Δ_i_	w_*i*_
Recapture rates (p)	state	state*Ad+Age*t	state*Ad+Age*t	128	31561.86	31817.86	0	0.99
	*c*			127	31575.12	31829.12	11.26	0.00
	state+*t*			156	31530.10	31842.10	24.24	0.00
	*t*			155	31544.65	31854.65	36.79	0.00
Survival rates (φ)	state	state*Ad+Juv+*t*	Age*t+state*Ad	100	31597.62	31800.00	0	0.99
		state*Ad+Age*t		128	31561.86	31820.23	20.23	0.00
		Age+*t*		99	31678.40	31878.78	78.78	0.00
		state*Ad+Juv		72	31792.08	31938.45	138.45	0.00
		state+*t*		99	31752.46	31952.83	152.83	0.00
Transition rates (ψ)	state	state*Ad+Juv+*t*	state*Ad+Juv+*t*	72	31624.86	31768.85	0	0.80
			Age+*t*	71	31629.78	31771.78	2.92	0.18
			state+*t*	71	31634.80	31776.80	7.95	0.00
			*t*	70	31651.85	31791.85	22.99	0.00
			state*Ad+Age*t	100	31597.62	31797.62	28.77	0.00

For survival and transition rate models, only the top five models are shown. See Table S1 for model notation. np, number of estimable parameters, dev, deviance; AIC, Akaike information criterion; Δ_i_, the AIC difference between the current model and the model with the lowest AIC value; w_*i*_, Akaike weight; state, state dependent rates; *c*, constant rates; *t*, time-dependant rates; Age, age-dependent rates; Ad, dependence of rates for adult birds (2+ years old) only; Juv, dependence of rates for first year breeders only; +, additive effect of variables; *, interactive effect of variables

**Table 2:** Summary results of the multievent mark–recapture analysis to estimate recapture, survival, and pair fidelity rates in male great tits

	Model Structure							
						
Parameter	p	φ	ψ		dev	AIC	Δ_i_	w_*i*_
Recapture rates (p)	state	state*Ad+Age*t	state*Ad+Age*t	128	26838.48	27094.48	0	0.89
	*c*			127	26844.74	27098.74	4.26	0.11
	state+*t*			156	26798.26	27110.26	15.78	0.00
	*t*			155	26804.68	27114.68	20.20	0.00
Survival rates (φ)	state	state*Ad+Juv+*t*	Age*t+state*Ad	100	26876.58	27076.58	0	0.99
		state*Ad+Age*t		128	26838.48	27094.48	17.9	0.00
		Age+*t*		99	26958.19	27156.19	79.62	0.00
		Age*t		127	26915.19	27169.19	92.62	0.00
		state*Ad+Juv		72	27050.88	27194.88	118.31	0.00
Transition rates (ψ)	state	state*Ad+Juv+*t*	state*Ad+Juv+*t*	72	26904.92	27048.92	0	0.99
			Age+*t*	71	26917.67	27059.67	10.75	0.00
			state+*t*	71	26922.61	27064.61	15.69	0.00
			state*Ad+Juv	44	26984.61	27072.61	23.69	0.00
			state*Ad+Age*t	100	26876.58	27076.58	27.66	0.00

For survival and transition rate models, only the top five models are shown. See Table S1 for model notation. np, number of estimable parameters, dev, deviance; AIC, Akaike information criterion; Δ_i_, the AIC difference between the current model and the model with the lowest AIC value; w_*i*_, Akaike weight; state, state dependent rates; *c*, constant rates; *t*, time-dependant rates; Age, age-dependent rates; Ad, dependence of rates for adult birds (2+ years old) only; Juv, dependence of rates for first year breeders only; +, additive effect of variables; *, interactive effect of variables

Application of the the MECMR to the three simulated datasets (with different recapture rates of birds in the “AD” state), revealed that our model correctly estimated (with very little bias) all of the input parameters (Table 3). More importantly, the MECMR model estimated pair fidelity rates far more accurately than did the traditional method in each of the three datasets, and this was particularly evident in the first simulated dataset where recapture was lowest (Table 3).

**Table 3:** Results of simulation analyses to assess the efficacy of the MECMR model to estimate state-dependent rates and the accuracy of both the new MECMR model and the traditional method to estimate pair fidelity rates

Method		MECMR			Traditional method
			
Simulations	State	p	φ	ψ	ψ
Dataset 1	“AS”	0.884	0.572	0.422	0.616
	“AD”	0.470	0.411	0.283	0.410
Dataset 2	“AS”	0.902	0.586	0.405	0.507
	“AD”	0.687	0.406	0.288	0.346
Dataset 3	“AS”	0.888	0.609	0.388	0.405
	“AD”	0.781	0.397	0.303	0.339

Estimates of the recapture rates (p), survival rates (φ), and fidelity rates (ψ) were obtained for birds breeding with the same partner as in the previous year (state “AS”) and those breeding with a different partner to the last year (state “AD”). Datasets were simulated using the following values (φ_AS_ = 0.6, φ_AD_ = 0.4; ψ_AS_ = 0.4; ψ_AD_ = 0.3; *p*_AS_ = 0.9, *c*_AS_ and *c*_AD_ = 0.73; *c*_AS/AD_ = 0.13, *l* = 0.92; see text for details) and with *p*_AD_ set to 0.5 (Dataset 1), 0.7 (Dataset 2), or 0.8 (Dataset 3).

## Discussion

Traditional means of estimating rates of pair fidelity in wild populations, where recapture rates are generally low and potentially heterogeneous can, in principle, lead to flawed biological inferences based on these rates. The novel multievent mark–recapture modeling framework we developed here overcomes these problems and thus allows robust and unbiased estimates of pair fidelity rates. The model has an additional advantage in allowing statistical exploration of heterogeneity in state transitions, and the covariation between these; something that is impossible using raw observations alone. We demonstrated the application of this framework to a 30-year great tit breeding data and showed that faithful birds display higher recapture rates and experience higher survival and fidelity rates compared with birds that change partners. The strong support for state-dependent parameters reveals that traditional estimates that ignore this variation are likely to be biased and also suggest that there might be large fitness costs associated with partner change in this system.

Our results provide the first evidence, to our knowledge, that pair status (i.e., breeding with the same partner or different partner in *t* − 1 and *t*) can influence recapture rates. While the importance of accounting for capture heterogeneity according to traits such as sex (Tavecchia et al. 2001; Lachish et al. 2011), age (Tavecchia et al. 2001; Bouwhuis et al. 2012), breeding status (Cam et al. 2002; Grosbois and Thompson 2005), dominance (Cubaynes et al. 2010), and infection status (Lachish et al. 2011) has already been emphasized, pair status as a source of capture heterogeneity has not previously been considered. The only related work of which we are aware is that of Klaich et al. (2011), who developed a likelihood-based model where capture rates of individuals in dyads (of a demographically closed population) varied between individuals that are associated and those that are not associated at the capture occasion *t*. The lower recapture rates for birds that changed partners between breeding seasons in this study might be a consequence of early breeding failure (e.g., Hannon and Martin 1996; Flynn et al. 1999), because birds were captured at a relatively late stage of the breeding cycle (when chicks were 6–14 days old). Alternatively, this pattern might result from differences in the chick provisioning rates or other behaviors of faithful pairs and pairs that had not bred together (Green 2002; Moody et al. 2005). Regardless of the underlying mechanisms, the existence of capture heterogeneity will bias estimates of pair fidelity obtained by methods that do not account for it, and argues against the use of traditional methods of estimating pair fidelity in wild populations.

A key advantage of the MECMR modeling framework developed in this study is that both fidelity and survival rates can be obtained in a single analysis and variation in these vital rates can be modeled as a function of pair status. These features allowed us to test hypotheses regarding the dynamics and fitness benefits of pair fidelity in the studied population. As predicted, our analyses revealed that faithful individuals experienced substantially higher survival rates than did individuals that changed partners between consecutive breeding seasons. Although it has been suggested that birds retain their partners so as to avoid potential survival costs (Ekman 1990; Pampus et al. 2005), we are aware of only one study that has provided empirical support for (or even tested for) this possibility (Nicolai et al. 2012). Our finding lends further support for possible survival cost associated with partner change.

Our results also agree with the common finding that pair fidelity rates are higher for adults than for yearlings (e.g., Pampus et al. 2005; Llambias et al. 2008). This pattern is usually attributed to yearling birds making more errors in initial mate choice (Choudhury 1995), placing higher importance on finding a better partner as they have more years to breed ahead (Choudhury 1995), and/or being left by their partners or expelled by another bird more often (Ens et al. 1993; Choudhury 1995) compared to adult birds. Traditional methods of analyzing pair fidelity rates utilize post hoc tests to assess such differences. The MECMR framework and model selection approach presented here is a more rigorous and powerful statistical method for such analyses. This approach also allowed us to demonstrate that birds breeding with the same partner at *t* − 1 and at *t*, were more likely to remain in the faithful state at *t* + 1, supporting the hypotheses that individuals that have found an acceptable partner (whether in terms of quality or compatibility, where compatibility can be genetic, Zeh and Zeh 1997; Hansson and Westerberg 2002; morphological, Black et al. 2007; behavioral, Spoon et al. 2006; or hormonal) tend to remain with them (Choudhury 1995). The finding that fidelity rates vary according to pair status (i.e., are state dependent) suggests that traditional estimates of pair fidelity rates may be biased and that inferences regarding the costs and benefits of pair fidelity based on them may be unreliable. The results of our simulation analyses strongly supported this suggestion, showing that traditional estimates of pair fidelity obtained in the presence of capture heterogeneity were inaccurate and biased.

### Utility and further applications of this MECMR modeling framework

The findings discussed above, while likely to be system specific, clearly highlight the potential of this new MECMR modeling framework to explore the causes, costs, and benefits of pair fidelity. The modeling framework easily accommodates the inclusion of external covariates (e.g., population density, sex ratio, or abiotic factors) and individual covariates (e.g., morphological or behavioral traits), which extend the range of hypotheses regarding the causes and consequences of pair fidelity that can be tested. Moreover, the MECMR model can be extended to include new states (e.g., “same partner/high quality territory”, “same partner/low quality territory” or “same partner/same territory”, “same partner/different territory”, etc.) to evaluate the relationship between fidelity and territory quality, or assess whether fidelity to a partner is driven by fidelity to a territory. Extension of the modeling framework would also allow state transitions to be modeled as higher order Markovian process (so-called memory models, as per Hestbeck et al. 1991), allowing the influence of past breeding experience on future pairing decisions to be analyzed (Rouan et al. 2009).

Our MECMR model was developed to assess rates of fidelity in pair-bonded individuals but can be applied to other dyadic associations where members of associating pairs are (i) not members of a breeding pair, for example to assess parent–offspring provisioning rates; (ii) individual and a particular location, for example to assess fidelity to a feeding patch or roosting site; (iii) an individual and a group this individual associates with, for example, to assess rates at which individuals change group membership; (iv) two groups of individuals, for example, to assess attachment of a certain type of pollinator to a certain type of plant. It is important to note, however, that care must be taken to avoid pseudo-replication. If the associating individuals belong to two discrete and distinguishable classes that remain unchanged over the study period (i.e., male and female, different species), then pseudo-replication can be avoided by analyzing association rates in the two classes separately (as was done in our analysis of pair fidelity in great tits). In other situations, reducing the length of the study period to ensure individuals remain in a single class (e.g., parent feeding a chick during one breeding season) can alleviate problems of pseudo-replication.

### Limitations of the MECMR approach

In all mark–recapture analyses, including MECMR models, mortality is confounded with permanent emigration. Our finding of lower survival rates for great tits that changed partners might thus be a consequence of higher rates of breeding dispersal for these individuals. A few previous studies on great tits have shown that breeding dispersal is generally lower for faithful birds compared to those that have changed partner (Andreu and Barba 2006; and references therein). Hence, we cannot discount the possibility that differential emigration drives the difference in apparent survival rates between faithful individuals and those that changed partners in our case study population. Where additional information on recoveries or resightings of marked individuals from a larger peripheral area is available, the joint analysis of such data in a MECMR framework can, under certain assumptions, allow for separating survival from emigration (Juillet et al. 2011). Another limitation of the MECMR model we present here is that estimates of pair fidelity are confounded with mortality of the focal individual's partner, which constrains interpretation of the results in terms of individual choice. An individual whose partner dies between breeding seasons must breed with a new partner even if the previous partner was the preferred one. We found that faithful great tits of both sexes (and hence, both members of faithful pairs) experienced higher survival rates than did individuals that changed partners. Consequently, members of faithful great tit pairs will have a greater opportunity to remain together from one breeding season to the next. Further development and extension of the MECMR framework to include additional states separating widowed and divorced individuals, could provide a means of investigating how two different strategies (stay with a partner or divorce) are adopted by individuals in the population in relation to different ecological and social factors. Nevertheless, if we assume that widowing rates are related to mortality rates, then we can estimate divorce rates from the vital rate estimates produced by the MECMR model (although only for individuals in the “AS” state). For example, in our great tit population, the divorce rate for females in state “AS” (*d*_AS_female_) can be calculated from their fidelity rate (ψ_AS_female_) and the mortality rate of males in state “AS” (1−ϕ_AS_male_):


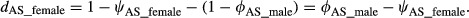


This is possible because the partners of “AS” females are “AS” males, for which we have mortality estimates. On the other hand, for individuals in the state “AD” we can only obtain approximate divorce rates, as we cannot tell at which rates their partners die (their partners can either be other adult birds in the state “AD,” or yearling birds, and these two classes have different mortality rates).

In conclusion, we have shown that, compared to previously used methods, the MECMR framework we have developed is not only able to provide parameter estimates accounting for capture heterogeneity and without discarding partial information, but also allows, either in its current form or using further extensions, an exploration of different evolutionary hypotheses on correlates, costs, and benefits of pair fidelity.

## Supporting Information

**Data S1.** Using the program E-SURGE for fitting model of pair fidelity.
