# Supplementary material for: A multievent approach to estimating pair fidelity and heterogeneity in state transitions
Source: Ecol Evol. 2013 Oct 4;3(13):4326–38. doi: 10.1002/ece3.729 (PMC3856734; doi:10.1002/ece3.729)

Supplement – Using the program E-SURGE for fitting model of pair fidelity

We have developed a multi-event mark-recapture model to assess annual rates of pair fidelity (i.e. breeding with the same partner as in the last year), partner change (i.e. breeding with the different partner to the last year partner), and survival, while explicitly accounting for imperfect detectability and heterogeneity in detection probabilities among individuals in different states (pair status). The model combines mark–recapture data with data from auxiliary breeding records to inform an individual's state (pair status).

The states considered in the model are:

'AS' (Alive with the Same partner), the focal individual is alive and breeding with its partner from the previous year;

'AD' (Alive with a Different partner), the focal individual is alive and breeding with a different partner to its partner in the previous year;

'D' (Dead), the focal individual is dead;

The events we can observe are:

- event 0 = the focal individual is not captured in the current breeding season (i.e. at *t*), its partner from the previous season (*t*-1) is either not captured at *t*-1 or *t* , or is captured breeding at *t* at an active nest with an unknown partner;
- event 1 = the focal individual is captured at *t*, and is breeding with its partner from *t*-1;
- event 2 = the focal individual is captured at *t* but is breeding with a different partner to that from *t*-1;
- event 3 = the focal individual is captured at *t* but it is not known whether its current partner is the same as the one from *t*-1;

- event 4 = the focal individual is captured at *t*, its current partner is not captured, and its partner from *t*-1 is captured at *t* but at a different nest (and hence is not breeding with the focal individual at *t*);
- event 5 = the focal individual is captured at *t*, its current partner is not captured, and its partner from *t*-1 is either not captured in *t* or was not known in *t*-1;
- event 6 = the focal individual is not captured at *t* (and hence its current partner is unknown), but its partner from *t*-1 is captured breeding with another individual at *t*.

The parameters estimated by the model are:

φ, survival probability;

ψ, transition probability, conditional on survival;

p, recapture probability (of the focal individual);

c, capture probability of focal's current (*t*) partner, conditional on the capture of the focal individual. This parameter incorporates information on the capture of the current partner and its identity in relation to the focal individual's *t*-1 partner. If *t*-1 partner is not known then, even if the current partner is captured, we cannot tell if it is the same as or different to *t*-1 partner. However, if the focal's current partner was breeding with an individual other than the focal individual in *t*-1, then this indicates partner change;

l, probability of capturing focal's *t*-1 partner at *t* (if *t*-1 partner is known) incorporating knowledge of its pairing status in *t*;

1. Fitting the pair fidelity model

After starting E-SURGE the first step is to load the dataset consisting of capture histories and any covariates that have been included. The top of the dataset (in the Headed format) we used is shown below, where ‘H:’ stands for capture history, ‘S:’ stands for number of birds with a specific capture history (in our case each row is a capture history for one bird only); ‘$COV:Mgp’ stands for the explanatory variable ‘Marking group’ :

```
 H:                                S:   $COV:Mgp
000003000000000000000000000000     1     Juv
000000036000000000000000000000     1     Ad
000000003600000000000000000000     1     Ad
000000360000000000000000000000     1     Juv
000003222000000000000000000000     1     Juv
000000300000000000000000000000     1     Juv
000000003000000000000000000000     1     Ad
```

As we have individuals that were captured as both yearlings and adults we include a covariate in the input file, which we labelled as ‘Mgp’ to indicate to which of the two Marking groups an individual belongs (individuals first captured as yearlings are coded with ‘Juv’, individuals first captured as adults are coded as ‘Ad’). Later we will use GEMACO code to obtain rates for the two age-classes we used in our study (yearlings and adults). When the data are loaded, a window asking you to choose covariates appears. Choose the first option (top left corner) by clicking the ‘Select’ button:

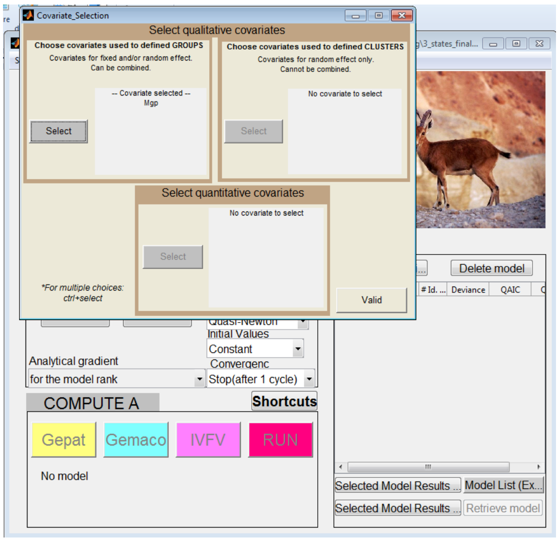

E-SURGE makes assumptions about the number of states and age classes. However, these need to be modified in our case. Change the number of states from 7 to 3 (i.e. ‘AS’, ‘AD’,

‘D’), and change the number of age classes to 2 by clicking the ‘Modify’ button on the main screen and then changing values in the corresponding boxes to look like this:

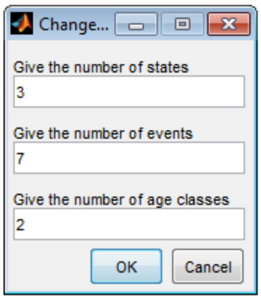

In E-SURGE, models are built in several stages. First, the GEPAT (Generator of Pattern of elementary matrices) allows specifying the initial state vector and the transition and event matrices. GEPAT matrices are row stochastic, so the sum of all cell probabilities in each row must add to one. Thus, one cell probability in each row will be calculated as the complement of the others. This cell is denoted with ‘*’. There are some cells with a structural probability being equal to zero. These cells are denoted as ‘-‘. After specifying the matrices, the GEMACO (Generator of Matrix of Constraints) interface is used to constrain the parameters of interest to vary according to the model we are building, or the hypotheses we are testing.

1.1 Specifying the pattern matrices using the GEPAT interface

In this step we specify which parameters of the model will be estimated and which will be calculated as the complement of the other parameters. We also define those parameters that correspond to impossible events or transitions (fixed to zero).

To activate the GEPAT interface, click on the GEPAT button at the left bottom corner of the main window.

The initial state vector

The first matrix to appear in GEPAT is the matrix of the initial state probabilities which is composed of a single row with two states. The 'Dead' state cannot appear as the initial state.

AS	AD	D

$$(\pi \quad 1-\pi \quad 0)$$

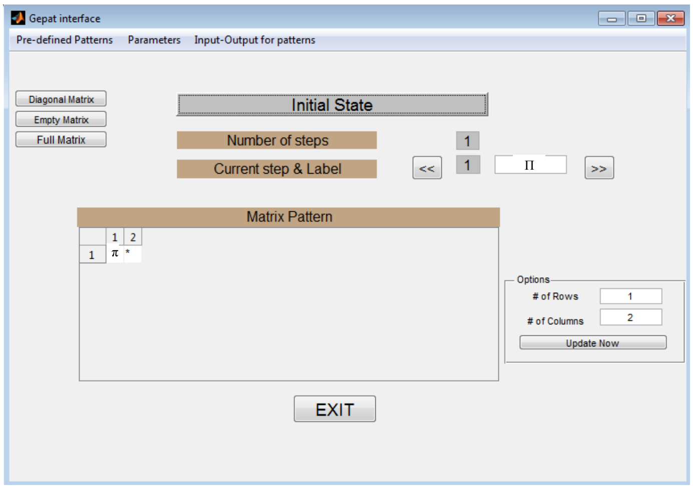

Later, at the IVFV stage, we have arbitrarily chosen to fix the probability of 'AD' to be 1 (i.e. the probability of 'AS' is then equal zero).

The matrices of survival and transition probabilities

We modelled the probability of transitioning between states as a two-step process composed first of the probability of survival over the yearly time interval and then the probability of transitioning among states (i.e. transitions among states are conditional on survival over the time-period). GEPAT initially offers only one step for transition. To increase the number of steps, enter '2' in the box right to the 'Number of steps'. We named the two transition matrices as 'survival' and 'transition'.

The rows of the survival matrix specify the possible underlying states at time *t*.

Thus, we specify the survival matrix as follows:

$$\begin{array}{c} \\ AS \\ AD \\ D \end{array} \begin{array}{c} \begin{array}{ccc} AS & AD & D \end{array} \\ \begin{pmatrix} \varphi & 0 & 1-\varphi \\ 0 & \varphi & 1-\varphi \\ 0 & 0 & 1 \end{pmatrix} \end{array}$$

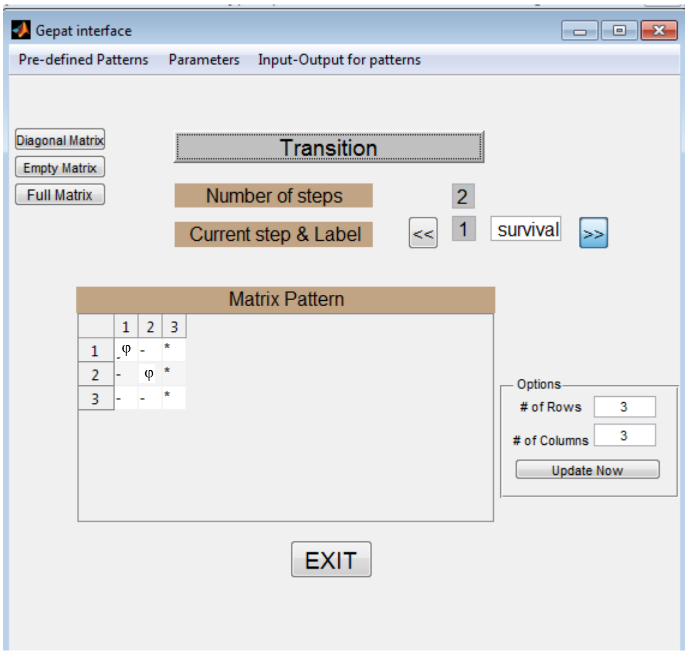

The rows of the transition matrix specify the possible states occupied at time *t*, while the columns specify the possible states occupied at time *t*+1.

Thus, we specify the transition probabilities as follows:

$$\begin{array}{c} \\ AS \\ AD \\ D \end{array} \begin{array}{c} \begin{array}{ccc} AS & AD & D \end{array} \\ \begin{pmatrix} \psi & 1-\psi & 0 \\ \psi & 1-\psi & 0 \\ 0 & 0 & 1 \end{pmatrix} \end{array}$$

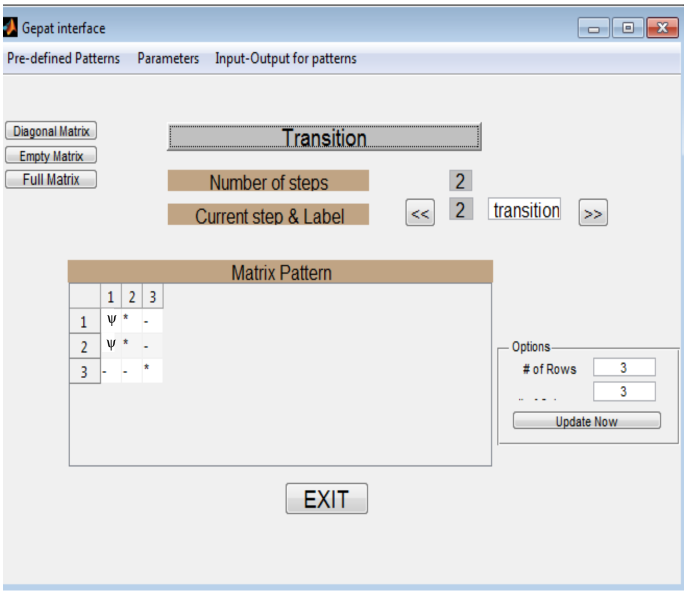

The event matrices

We define three different event matrices (event probabilities are modelled in three steps; see Methods section for details). To increase the number of event matrices enter '3' in the box right to the 'Number of steps'. We called these matrices as [P] (focal bird recapture), [P2] (capture of the current partner), and [P3] (last-year partner recapture).

The matrix of **Step 1** describes the recapture probability of the focal individual ('FC' denotes the focal bird is captured, 'FNc' denotes a bird is not captured, superscript symbols after 'FC' and 'FNc' specify in which underlying state the focal bird is):

$$\begin{array}{c} \\ AS \\ AD \\ D \end{array} \begin{array}{c} \begin{array}{ccccc} FNc^{AS} & FNc^{AD} & FNc^{D} & FC^{AS} & FC^{AD} \end{array} \\ \begin{pmatrix} 1-p & 0 & 0 & p & 0 \\ 0 & 1-p & 0 & 0 & p \\ 0 & 0 & 1 & 0 & 0 \end{pmatrix} \end{array}$$

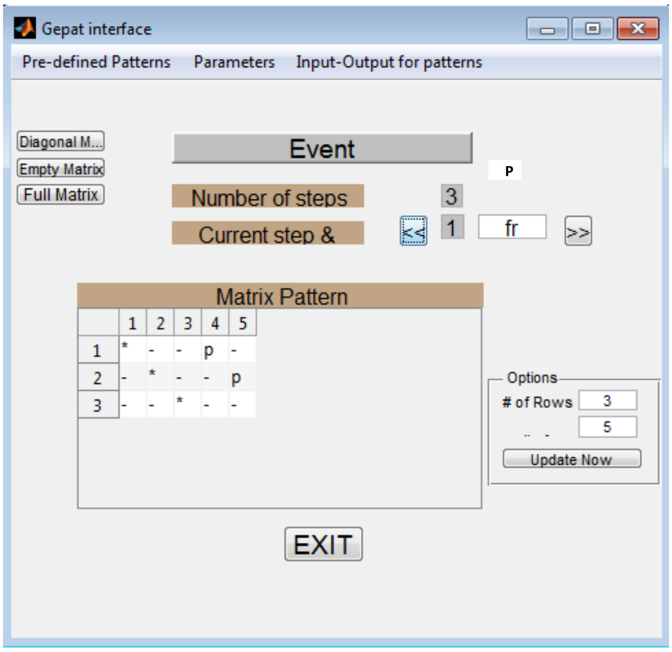

The matrix of **Step 2** describes the probability of capturing the focal individual's current partner at *t* and knowing its identity in relation to the focal individual's *t*-1 partner, conditional on the capture of the focal individual at *t*. The rows in this matrix correspond to the columns of the preceding matrix in **Step 1** (when modelling event probabilities, in each subsequent step the columns of the previous matrix become the rows of the next matrix). 'PC' denotes that current partner is captured; 'PNc' denotes that current partner is not captured; 'PNn' denotes current partner is captured but it is not known if it is the same or different to *t*-1 partner. Symbols in superscrips after 'PC', 'PNc', and 'PNn' specify in which underlying state a focal bird is.

PNc^AS PNc^AD PNc^D PC^AS PC^AD PNn^AD,AS PNc^AS PNc^AD

$$\begin{pmatrix} 1 & 0 & 0 & 0 & 0 & 0 & 0 & 0 \\ 0 & 1 & 0 & 0 & 0 & 0 & 0 & 0 \\ 0 & 0 & 1 & 0 & 0 & 0 & 0 & 0 \\ 0 & 0 & 0 & c & 0 & c & 1-\sum c & 0 \\ 0 & 0 & 0 & 0 & c & c & 0 & 1-\sum c \end{pmatrix}$$

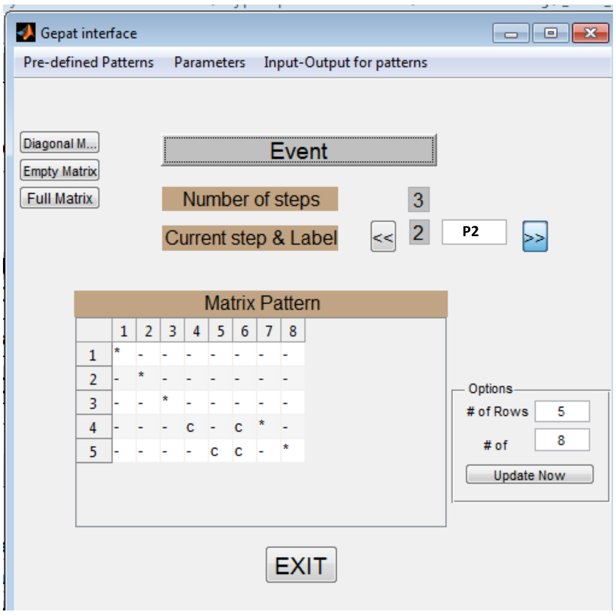

The matrix of **Step 3** describes the probability of capturing the focal individual's *t*-1 partner if it was known in *t*-1) in the current year (*t*), incorporating information on its pairing status in the current year. Rows correspond to the columns of **Step 2**. The column numbers correspond to the event codes found in the capture histories.

0 1 2 3 4 5 6

$$\begin{pmatrix} 1 & 0 & 0 & 0 & 0 & 0 & 0 \\ 1-l & 0 & 0 & 0 & 0 & 0 & l \\ 1-l & 0 & 0 & 0 & 0 & 0 & l \\ 0 & 1 & 0 & 0 & 0 & 0 & 0 \\ 0 & 0 & 1 & 0 & 0 & 0 & 0 \\ 0 & 0 & 0 & 1 & 0 & 0 & 0 \\ 0 & 0 & 0 & 0 & 0 & 1 & 0 \\ 0 & 0 & 0 & 0 & 1-l & l & 0 \end{pmatrix}$$

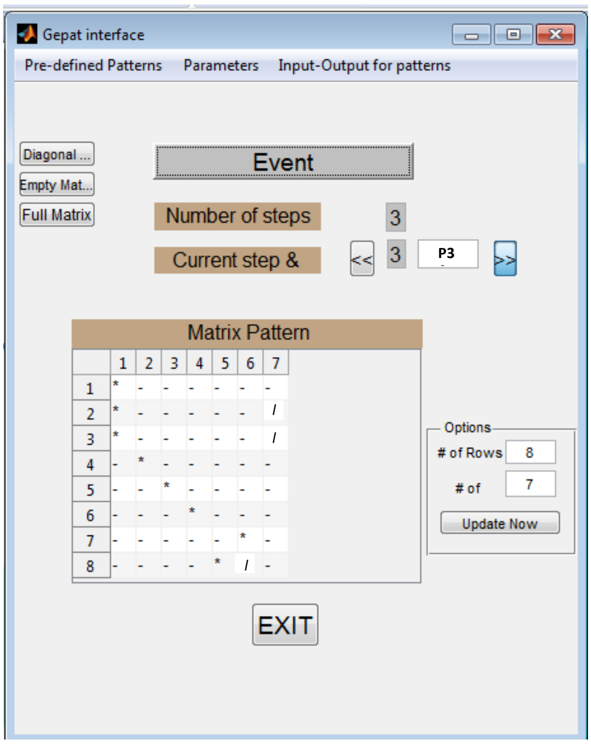

1.2 Specifying the model (defining shortcuts and using GEMACO interface)

To specify models, we have to define how parameters vary over time, groups, age classes, etc. To do this we use GEMACO interface to create a design matrix for each type of parameters.

First, we have to create a shortcut for age classes (yearling and adult birds). To do this click on the ‘Shortcuts’ button on the main E-SURGE screen. In our input file we have already define explanatory variable ‘Mgp’ that can take values ‘Ad’ and ‘Juv’ depending on whether a bird was first caught as an adult or a yearling. This is shown in the ‘Shortcuts’ interface as:

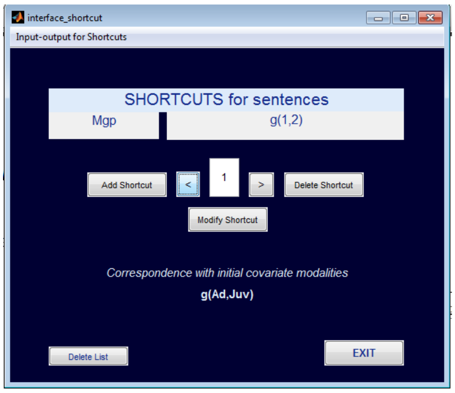

Next, click on the ‘Add shortcut’ indicator to create a new shortcut. Create two shortcuts ‘Ad’ and ‘Juv’ by typing a specific shortcut formula as shown below (for details on the meaning of GEMACO code please refer to the ESURGE manual, Choquet 2011):

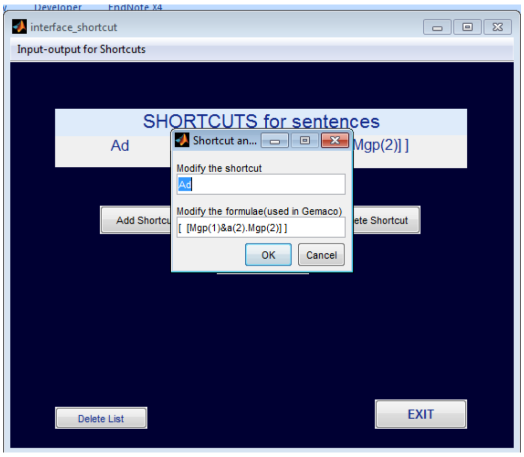

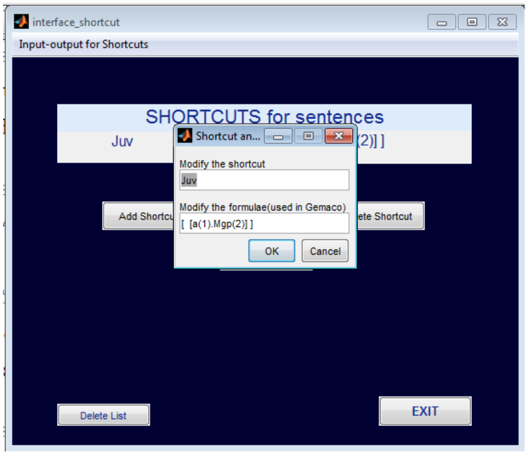

When the shortcuts are defined, click the ‘EXIT’ button to exit the shortcut interface. Click the ‘GEMACO’ button to enter the GEMACO interface. In the GEMACO interface a syntax specifying variation of each parameter (independently of each other) is entered in the ‘Model definition’ window.

We constrain the initial state (at the IVFV stage we fix the probability of AD to be 1) to be constant over the study period, so leave the window blank and click on the ‘Initial state’ button to get at the ‘Transition’ screen.

To allow survival and transition probabilities to depend on both current state and age, and to be time varying, write the formula ‘**from.Ad+Juv+t**’ in the ‘Model definition’ box as shown below (shown for the first step, i.e. survival only, but the same GEMACO code is entered in both windows).

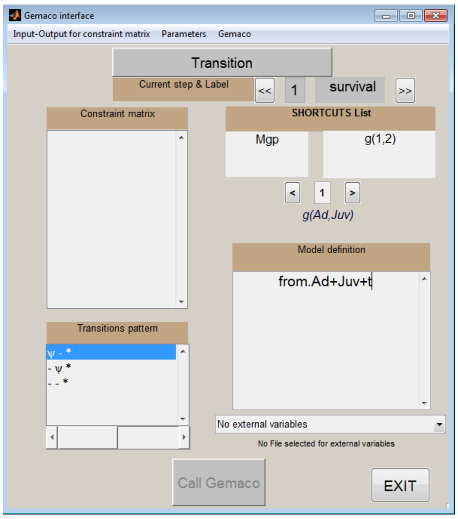

Click on the top ‘Transition’ button to progress to the ‘Event’ screen.

Multi-event models condition on the first encounter of each individual. This means that the event ‘not encountered’ becomes possible only after an individual has been caught. Thus, the event probabilities at the time of the first encounter must be treated separately from the subsequent event probabilities (and later fixed to one). In E-SURGE this is achieved by using the keywords ‘**firste**’ (stands for ‘first encounter’) and ‘**nexte**’ (‘next encounters’). To specify that event probabilities will vary with state and to allow for fixing the probability of the first encounter, write the following syntax into the ‘Model definition’ window: ‘**firste+nexte.to**’. We will fix the probability of the first encounter to be one at the later (IVFV) stage.

In the **Step 2** of the event probabilities we specify the ‘capture’ probability of the focal individual’s partner to depend on the current state. In our case, the capture probabilities in columns 4 and 5 of the event matrix represent true capture probabilities of the current partner, while the probabilities in the column 6 represent the combined probabilities of the capture of the current partner and the probability of knowing it’s relation to the last-year partner. To differentiate these, we write (4 5, 6) in the brackets after ‘**to**’. This forces the capture probabilities in the columns 4 and 5 to be equal and different form the capture probability in the column 6. The final syntax looks like this: ‘**firste.to(4 5,6)+nexte.to(4 5, 6)**.

Finally, in **Step 3** of the event probabilities we specify the probability of the recapture of the last-year partner. At the first capture of the focal individual the event 4 is not possible (i.e. probability of event 5, as the only other option, is 1) as the partner from the last year cannot be known for individuals captured for the first time, and consequently it cannot be captured in the current year. Thus, we need to allow for fixing the event 5 to equal 1 at the time of the first encounter later in the IVFV stage. To achieve this write the syntax ‘**firste.from(8)+nexte.from(8)+from(2,3)**’ in the ‘Model specification’ window.

After specifying how the parameters of the initial state vector, and survival-transition and event matrices vary, we need to create the design matrices. This is done by clicking the ‘Gemaco’ item in the top menu and choosing ‘call GEMACO (all phrases)’ option:

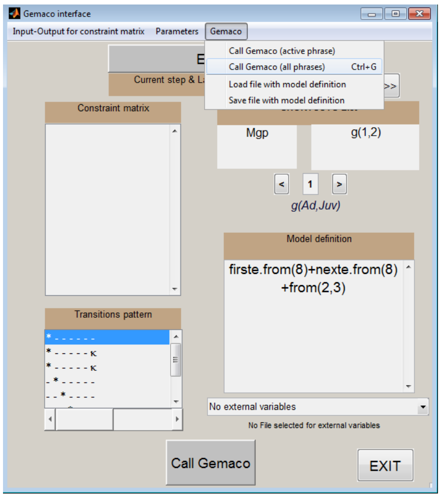

Now, all the model structures are specified and the design matrices appear in the left window of each screen of the GEMACO interface (shown below, only for the **Step 3**). To return to the main window click the EXIT button.

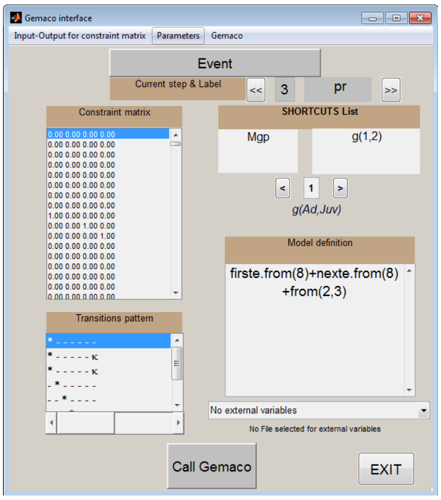

1.3 Specifying the initial and fixed values using the IVFV interface

In the ‘Advanced Numerical Options’ part of the main window click the box left to ‘Compute C-I (Hessian)’ so that standard errors can be obtained. Choose ‘Multiple random’ option from the drop-down menu below the ‘Initial values’, and type the number of initial random values. This specifies the way the initial values of the optimisation procedure are generated and assists with model convergence issues.

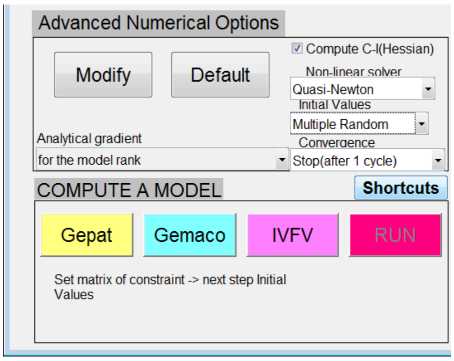

Press the IVFV button to enter the interface.

For the initial state probabilities we have arbitrary chosen to fix the probability of ‘AD’ to be 1 (i.e. the probability of ‘AS’ is then equal zero).

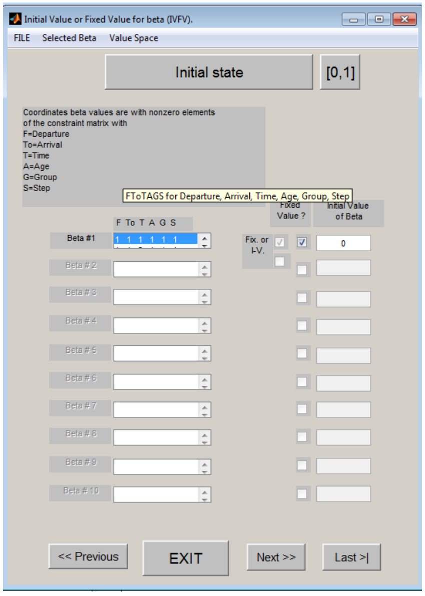

Click on the top ‘Initial state’ button to arrive at the ‘Transition’ screen. There is no need to fix the values for the transition probabilities, so leave the screen at its default state.

As discussed above, the probability of the first encounter of the focal individual equals 1, and the probability of capturing focal’s last-year partner equals 0 in the current year (i.e.

probability of not capturing it equals 1). To ensure this, fix the event probabilities as shown below:

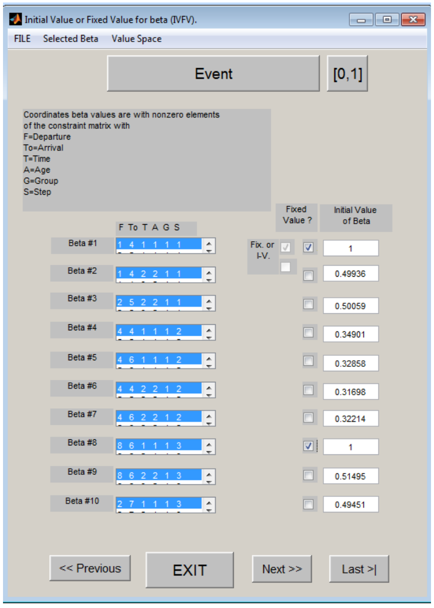

After specifying all the fixed values press the EXIT button. Press the RUN button of the main menu to run the model.

Literature

Choquet, R., E. Nogue. 2011. E-SURGE 1.8 User’s Manual. CEFE, Montpellier, France.

Table 1S Model notation describing the recapture, survival and transition rate models included in the candidate list for multi-event mark–recapture modelling of survival and pair fidelity rates in great tits.

Recapture	Survival	Between-state transition
c	c	c
t	t	t
state	Age	Age
state+t	state	state
	Age+t	Age+t
	state+ t	state+t
	Age*t	Age*t
	state*Ad+Juv	state*Ad+Juv
	state*Ad+Juv+t	state*Ad+Juv+t
	state*Ad+Age*t	state*Ad+Age*t
		state*t

Figure 1S Recapture rates for pair faithful (state ’AS’, black bars) and partner changed (state ‘AD’, grey bars) females and males with upper 95% CI.

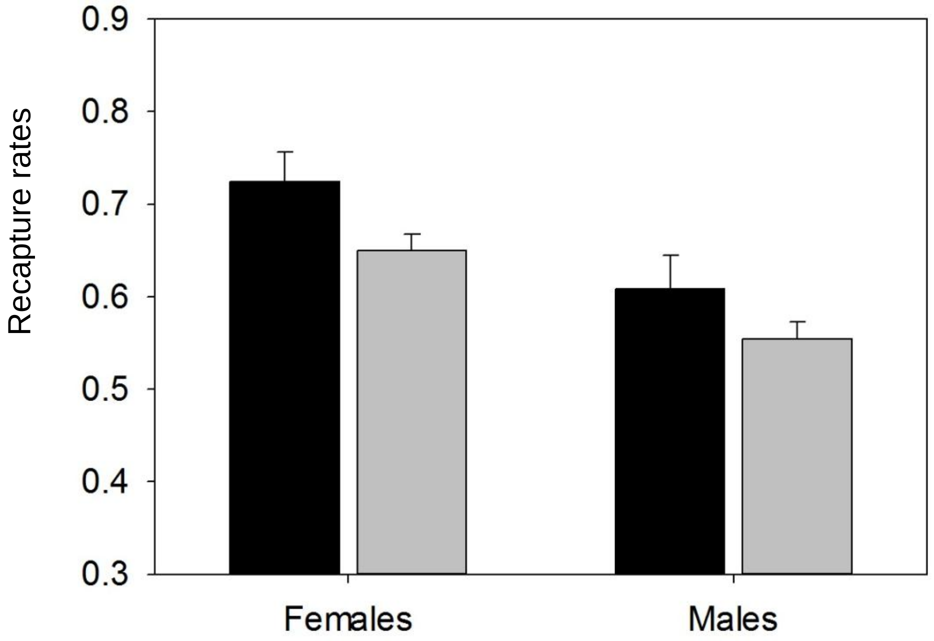

Supplement: Supplementary file 1 [file ece30003-4326-SD1.pdf]
